# Urban growth modelling and social vulnerability assessment for a hazardous Kathmandu Valley

**DOI:** 10.1038/s41598-022-09347-x

**Published:** 2022-04-12

**Authors:** Carlos Mesta, Gemma Cremen, Carmine Galasso

**Affiliations:** 1 Understanding and Managing Extremes (UME) Graduate School, Scuola Universitaria Superiore IUSS Pavia, Pavia, Italy; 2 Department of Civil, Environmental and Geomatic Engineering, University College London, London, UK

**Keywords:** Sustainability, Natural hazards

## Abstract

In our rapidly urbanizing world, many hazard-prone regions face significant challenges regarding risk-informed urban development. This study addresses this issue by investigating evolving spatial interactions between natural hazards, ever-increasing urban areas, and social vulnerability in Kathmandu Valley, Nepal. The methodology considers: (1) the characterization of flood hazard and liquefaction susceptibility using pre-existing global models; (2) the simulation of future urban built-up areas using the cellular-automata SLEUTH model; and (3) the assessment of social vulnerability, using a composite index tailored for the case-study area. Results show that built-up areas in Kathmandu Valley will increase to 352 km^2^ by 2050, effectively doubling the equivalent 2018 figure. The most socially vulnerable villages will account for 29% of built-up areas in 2050, 11% more than current levels. Built-up areas in the 100-year and 1000-year return period floodplains will respectively increase from 38 km^2^ and 49 km^2^ today to 83 km^2^ and 108 km^2^ in 2050. Additionally, built-up areas in liquefaction-susceptible zones will expand by 13 km^2^ to 47 km^2^. This study illustrates how, where, and to which extent risks from natural hazards can evolve in socially vulnerable regions. Ultimately, it emphasizes an urgent need to implement effective policy measures for reducing tomorrow's natural-hazard risks.

## Introduction

The world’s population continues to grow and is expected to reach 9.7 billion in 2050^1^. By this time, 68% of the global population will be living in cities, with nearly 90% of urban growth occurring in the least developed regions of Asia and Africa. In addition, low-income and lower-middle-income countries are projected to experience the fastest urbanization rates in the coming decades^2^. The physical extent of urban areas is growing even at faster rates than the corresponding population^3,4^. By 2030, cities are expected to triple the amount of land used in 2000, with much of the increase occurring in relatively undisturbed biodiversity hotspots^5^. The spatial expansion of urban areas will have severe implications for energy consumption, climate change, and environmental degradation. Therefore, there is an urgent need to develop appropriate strategies for managing urban growth in an informed way^6^.

Effective development planning for urban areas should pay particular attention to disaster risk management and reduction, and climate change adaptation^2^. In 2018, 60% of cities with 500,000 or more inhabitants were highly exposed to at least one of six natural hazards (cyclones, floods, droughts, earthquakes, landslides, and volcanic eruptions), and this number is growing^7^. Moreover, it has been evidenced that poor people disproportionally suffer the effects of natural-hazard-induced disasters due to lower socio-economic resilience (e.g., low-income levels, less support from financial instruments, such as insurance, and social protection schemes)^8^. These primary concerns have been formally remarked in international agreements such as the 2030 Agenda for Sustainable Development^9^, the Paris Agreement on Climate Change^10^, and the 2015-2030 Sendai Framework for Disaster Risk Reduction^11^.

Current natural-hazard risk assessments exhibit gaps that limit their ability to support policymaking and planning decisions to reduce disaster risk in tomorrow's world^12,13^. Risk (defined as the convolution of hazard, exposure, and vulnerability) grows under natural and human influences. For instance, exposure is constantly evolving, driven by population growth and socio-economic development. Also, as population and economic activity increase, the proportion of urban land becomes larger^14^. Despite these facts, many disaster risk methodologies still use a static view of exposure (i.e., unchanged exposed elements, for which physical vulnerability is not time-dependent)^15^. While some researchers have attempted to model natural-hazard risk from a future-focused perspective^16–19^, most frameworks neglect the dynamics and interactions between risk components. Traditional approaches examine the impact of single natural hazards, overlooking relationships/interactions between multiple hazards and human activity^20,21^. Additionally, in contrast with the large effort that is typically dedicated to modelling physical vulnerability in natural-hazard risk assessments, there is less focus on assessing social vulnerability and how communities respond to extreme events^22,23^. There is a necessity to address these methodological flaws in the literature and "move instead towards risk assessments that can guide decision-makers towards a resilient future"^14^.

This research contributes to the required effort by portraying a dynamic representation of risk from natural hazards that accounts for social vulnerability. In particular, this paper examines the relationships between natural hazards, urban growth, and social vulnerability in Kathmandu Valley, Nepal. Firstly, the footprints on flood hazard and liquefaction susceptibility are obtained from pre-existing global models^24,25^. Secondly, the cellular-automata SLEUTH (an acronym for Slope, Land use, Excluded areas, Urban extent, Transportation, Hillshade)^26^ model is applied to simulate Kathmandu Valley's urban growth at 2050. And thirdly, the Social Vulnerability Index (SoVI)^22^, adapted to account for Nepal's specific context, is employed to quantify social vulnerability in Kathmandu Valley. The results on spatially overlapping hazards, urban growth, and vulnerability help to identify critical locations where disaster risk is likely to increase drastically in the future. These locations require particular attention and should be prioritized in regional policy on disaster risk management.

## Materials and methods

### Study area: Kathmandu Valley

**Figure 1:**
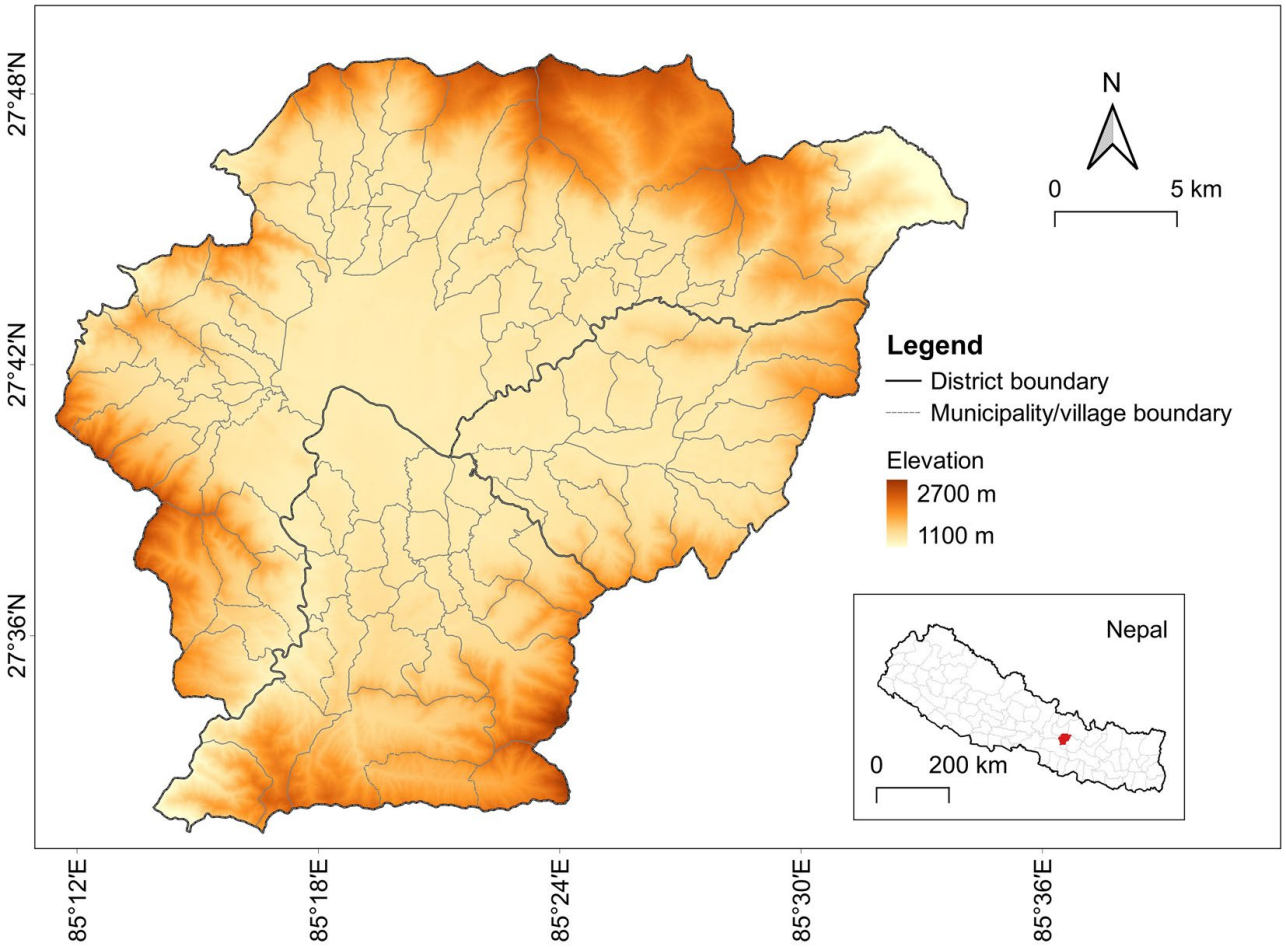
Physical and administrative map of Kathmandu Valley.

Nepal is among the least urbanized countries of Asia, but is projected to be one of the ten fastest urbanizing nations in the world over the 2018–2050 period^2^. Nepal's rapid urban growth has resulted from multiple urban transitions (spatial, demographic and economic). While urbanization is gaining pace in various regions of Nepal, Kathmandu Valley represents the "hub" of urban development in the country^27^. Geographically, Kathmandu Valley is surrounded by the Himalayan mountains and lies within the Bagmati river watershed. The valley extends from 27°49′4″ to 27°31′42″ latitude and from 85°11′19″ to 85°33′57″ longitude, accounting for a total spatial extent of 721 km^2^. Administratively, Kathmandu Valley encloses the entire Bhaktapur and Kathmandu districts and approximately 50% of the Lalitpur district. The valley contains five municipal areas and several municipalities and rural municipalities (formerly named village development committees, or VDCs), Fig. 1. The current population in Kathmandu Valley is estimated to be 3.3 million and is projected to reach 3.8 million by 2031^28^.

Geological (e.g., earthquakes, landslides) and hydro-meteorological hazards (e.g., floods, droughts) continuously threaten Nepal's development gains. According to the Global Climate Risk Index^29^, Nepal was among the ten countries most affected by extreme weather events over the 2000–2019 period. In addition, Kathmandu Valley is considered to be the urban area that is most at risk from seismic activity, worldwide^30^. Some recent natural-hazard events have produced devastating losses in Nepal, underlining its high vulnerability. For example, the 2015 Gorkha Earthquake caused over USD 7 billion in economic losses, 9,000 deaths, and 22,300 injuries^31^. The earthquake also triggered several liquefaction events across Kathmandu Valley^32^. Two years later, the 2017 monsoonal precipitation struck 80% of the Terai region and surrounding districts. The resulting flooding caused USD 584.7 million in damage, 160 deaths, and 45 injuries^33,34^. The intensity of hydro-metrological hazards is expected to increase in the future due to the impact of climate change. For instance, monsoonal precipitation for Nepal is projected to rise by 3–8% in the medium-term (2016–2045) and 9–14% in the long-term (2036–2065)^35^.

### Hazard modelling

We used two pre-existing global datasets to characterize flood hazard and liquefaction susceptibility in Kathmandu Valley. The flood maps (90 m resolution) and liquefaction map (1.2 km resolution) were resampled to 30 m using the nearest neighbor method^36^, to match the spatial resolution of SLEUTH outputs.

#### Flood hazard

To represent flood hazard, we used the high-resolution Fathom-Global model^24^, which accounts for both fluvial (riverine) and pluvial (surface water) inundation. This model uses the Multi-Error-Removed Improved-Terrain (MERIT) digital elevation model^37^ and MERIT Hydro^38^ as topography and hydrography datasets, respectively. The modelling framework considers a 2D shallow-water formulation to explicitly simulate flood wave propagation and uses a regionalized flood frequency analysis^39^ to estimate river discharge. The Fathom-Global model provides maps of flood extents and flood depths for multiple return periods (from 1:5 year to 1:1000 year).

**Figure 2:**
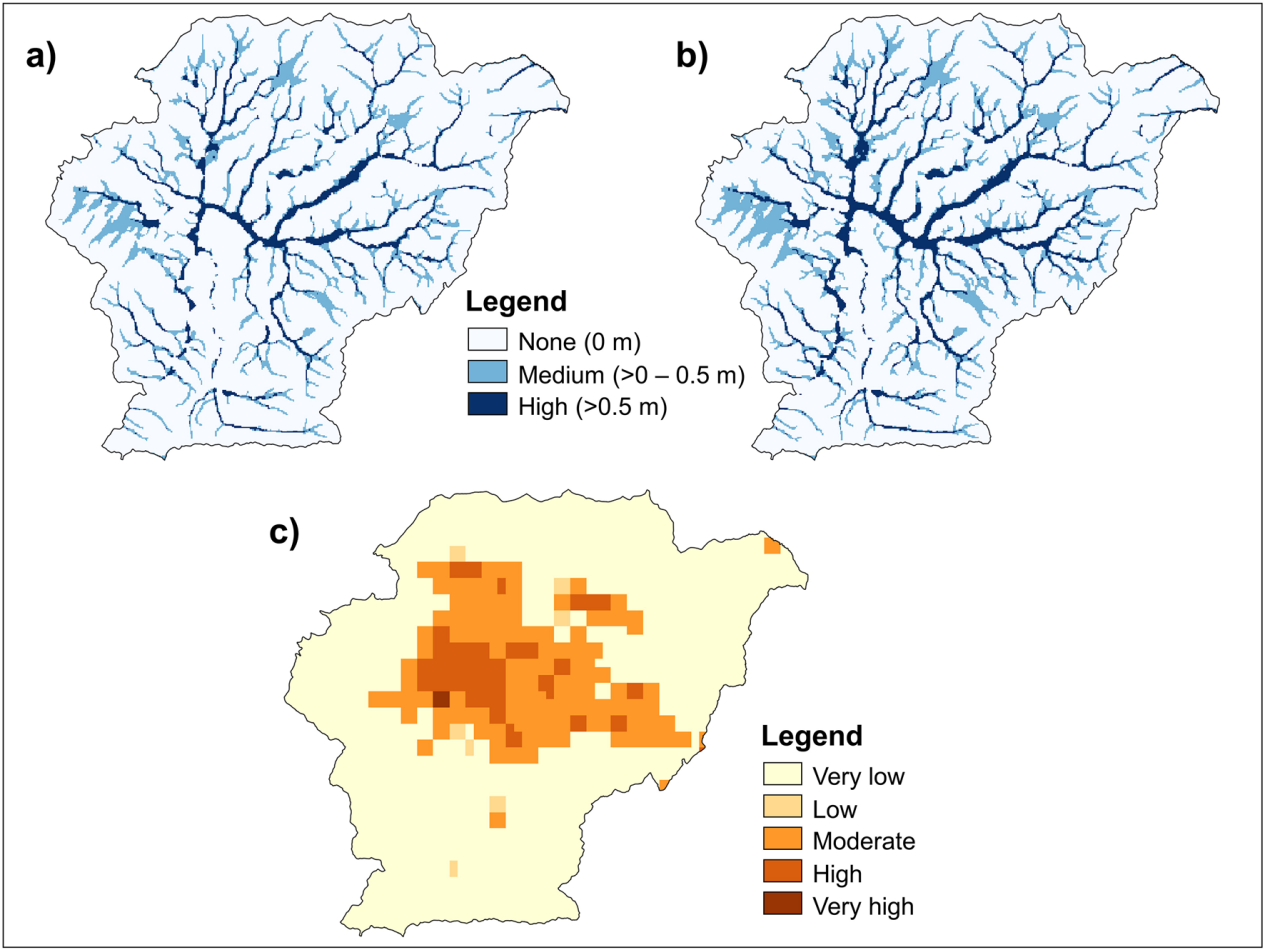
Characterization of considered natural hazards (**a**) Fluvial-pluvial 100-year flood hazard map; (**b**) Fluvial-pluvial 1000-year flood map; (**c**) Liquefaction susceptibility map.

In this study, we characterized two cases of flooding occurrence. The first case incorporates the undefended flood map with a 100-year return period (i.e., 1% probability of occurring in any given year) to represent today’s hazard condition. The 100-year return period map is the one most commonly used by decision-makers (e.g., to identify flood risk zones in the United States)^40^. The second flood-occurrence case reflects a worst-case situation, approximately capturing the exacerbation of flooding due to climate change. We selected the undefended flood map for the 1000-year return period to represent this case. Note that urbanization effects on flood hazard (as a result of increased runoff during rainfall events) are not explicitly accounted for by the Fathom-Global model, and are therefore neglected in our analyses. Individual flood maps were combined (by taking the maximum depth from the individual maps) into aggregated hazard maps that represent fluvial-pluvial flooding for each return period, in line with the method of Tate et al.^41^. The combined-map flood hazard levels were then categorized according to ranges of water depth as none (0 m), medium (> 0–0.5 m), or high (> 0.5 m) (Fig. 2).

#### Liquefaction susceptibility

To represent liquefaction, we used a global liquefaction susceptibility map^25^. This dataset was created by adopting the geospatial prediction models for inland and coastal regions proposed by Zhu et al.^42^. These empirical models relate earthquake-induced ground-motion intensity measures (e.g., peak ground acceleration, or PGA) with geospatial parameters (i.e., 30 m averaged shear-wave velocity or V_S30_, rivers, ground water, precipitation, land mass) that contribute to liquefaction susceptibility. The models were calibrated on 27 earthquake events and have demonstrated a reliable predictive capacity at high resolutions^43^. The map categorizes liquefaction susceptibility into five classes: very low, low, moderate, high, and very high (Fig. 2). Very low susceptibility corresponds to locations with V_S30_ > 620 m/s^44^.

It is worth noting that liquefaction susceptibility does not equal liquefaction hazard. Liquefaction susceptibility measures the degree to which a site may be potentially affected by liquefaction, based on the soil/geologic conditions and with no earthquake-specific information. Liquefaction hazard is usually defined as a combination of the triggering event (i.e., ground shaking from earthquakes with a specified return period) and susceptibility^45^, and is more consistent with our characterization of flooding. However, hazard-map ground-shaking intensities do not vary significantly across the relatively small area of Kathmandu Valley. For instance, according to the Global Earthquake Model (GEM) Global Mosaic of Hazard Modules^46^ (OpenQuake files available at https://github.com/nackerley/indian-subcontinent-psha, last accessed October 2021), maximum variations across Kathmandu Valley are approximately 13% and 9% for PGA with 10% probability of exceedance in 50 years and PGA with 2% probability of exceedance in 50 years, respectively. For this reason, we considered liquefaction susceptibility instead of liquefaction hazard or, ultimately, seismic hazard.

### Urban growth modelling with SLEUTH

Substantial advances in remote sensing technologies and spatial modelling have enabled the creation of worldwide spatial datasets on human settlements that can be used in natural-hazard risk modelling. These datasets are generally derived from satellite imagery and census data and vary in spatial resolution. For instance, the Global Human Settlement Layer (GHSL), produced by the European Joint Research Centre, is a global, fine-scale, and open dataset that depicts built-up surfaces and population distributions for 1975, 1990, 2000, and 2014 epochs^47^. The potential of the GHSL for natural-hazard risk modelling and disaster risk reduction has been demonstrated within the Atlas of the Human Planet 2017: Global Exposure to Natural Hazards^48^, which reveals the changes of exposure to six natural hazards (earthquakes, volcanoes, tsunamis, floods, tropical cyclone winds, and sea-level surge) at continental and country levels. Other spatial datasets on human population distribution, including Landscan^49^, WorldPop^50^ or the High Resolution Settlement Layer^51^, have been used for similar purposes and at various scales^52–57^.

Due to the availability of several land-use and land-cover (LULC) models, researchers have appropriate tools to make realistic future projections on land-use change and urban growth and investigate their various impacts (e.g., air pollution, soil degradation, food security)^58^. From many LULC modelling approaches identified in the literature (e.g., agent-based, artificial neural networks, cellular-automata, Markov chains)^59^, cellular-automata are among the most popular due to their simplicity, flexibility, and ability to integrate the spatial and temporal dimensions of urban growth^60^. In particular, SLEUTH is a mature cellular-automata model that uses historical maps to probabilistically determine future land-use. SLEUTH has been applied to simulate urban expansion in very heterogeneous areas at local, regional^61–67^ and global scale^68^. In addition, SLEUTH has been recently employed to assess future exposure to earthquakes in Jakarta, Metro Manila and Istanbul^19^ and future exposure to flooding in Helsinki^69^ and Shenzen^70^. LULC models have also been coupled with distinct modelling frameworks to identify other vulnerabilities (not related with natural-hazards occurrence) and suggest policies for tackling undesirable outcomes of urban sprawl development. Some examples include an urban growth scenario analysis to assess the impact of farmland preservation policies in Huangmei County, China^71^; the derivation of smart urban growth policy scenarios to mitigate the urban heat island effect in Brisbane, Australia^72^; and an urban growth scenario analysis to find optimal land-use strategies to improve ecosystem services (e.g., carbon storage, water yield, nitrogen export, habitat quality, food supply) in the Atlanta Metropolitan area, United States^73^.

In this study, we modeled a business-as-usual scenario, which considers future urban expansion to be an extension of historical urban growth. Under this scenario, future urbanization in Kathmandu Valley is assumed to occur without restriction, apart from in some areas that impose environmental constraints (i.e., water bodies, green areas, steep slopes). From a forward-looking risk perspective, the selected scenario represents the worst-case (i.e., most conservative, non-risk-informed) situation for investigating the consequences of coupling evolving exposure, natural hazards and vulnerability.

#### Data collection and processing

SLEUTH is an acronym for the six inputs that the model requires in raster format: Slope, Land-use, Excluded areas, Urban extent, Transportation, and Hillshade. We obtained the slope map by calculating the percentage terrain slope from the Shuttle Radar Topography Mission (SRTM) 1 Arc-Second Global (30 m resolution) digital elevation model (available at https://earthexplorer.usgs.gov/, last accessed June 2021). Cells having slope values higher than 35% were prevented from becoming urbanized, according to observed land development in Kathmandu Valley and similar to previous studies^74,75^. We also derived the hillshade map from the SRTM 1 Arc-Second Global data. The hillshade map is only used to visualize the SLEUTH outputs, and it does not influence the urban growth calculations. Land-use inputs were not needed, because we only focused on estimating urban growth. We extracted the water bodies, the airport runway and small green areas from OpenStreetMap (available at https://openstreetmap.org/, last accessed June 2021) to generate the excluded map (i.e., areas prohibited from urbanization).

**Figure 3:**
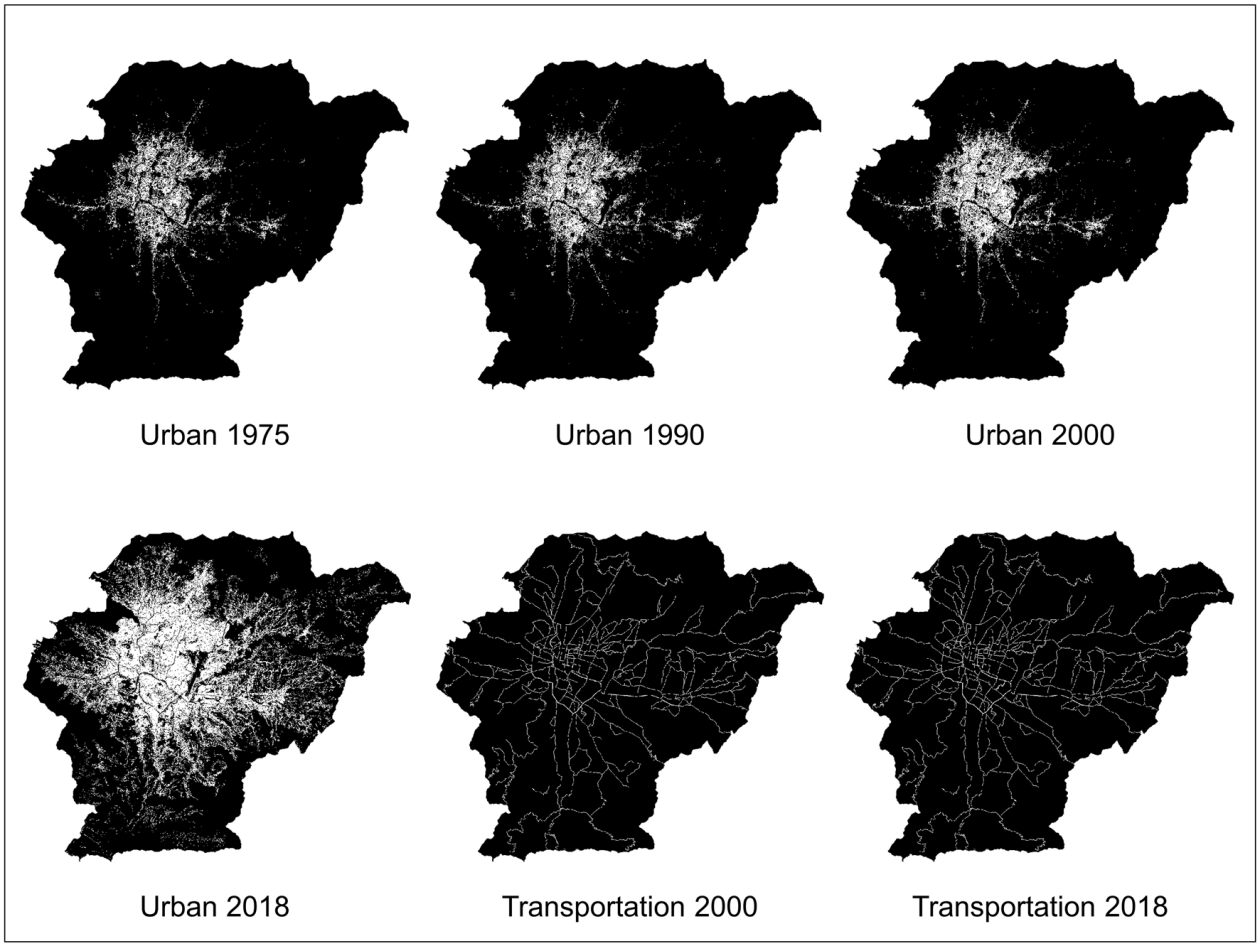
Urban extent and Transportation input data for SLEUTH.

The SLEUTH model calibration relies on at least four urban extents corresponding to different years (shown in Fig. 3), which are used as control points. For these inputs, we used the built-up areas of Kathmandu Valley in 1975, 1990, 2000, and 2018 from the urban maps of the GHSL. The built-up areas reported by GHSL are "areas where buildings can be found"^76^, regardless of their permanency. This definition allows for the inclusion of informal settlements, slums, and other temporary settlements. The 1975–1990–2000 urban maps were obtained from the GHS-BUILT-R2018A dataset (30 m resolution)^77^, while the 2018 urban map was taken from the GHS-BUILT-S2-R2020A dataset (10 m resolution)^78^. As the GHS-BUILT-S2-R2020A delineates the presence of built-up areas in a probability grid, we binarized the map (i.e., converted the map to urban/non-urban classes) using the suggested probability threshold of 0.2 (i.e., land was deemed to be urban if the probability is equal or greater than 0.2, otherwise land was categorized as non-urban)^79^. Additionally, we modified the class of a few pixels for consistency with the excluded map (e.g., we relabelled pixels located in the airport runway as non-urban).

SLEUTH relies on at least two transportation maps corresponding to different periods (shown in Fig. 3). For these inputs, we used 2000 and 2018 road maps of Kathmandu Valley, which were generated as follows. We extracted the trunk, primary, secondary, and tertiary road classes of OpenStreetMap for the current year and then consulted high-resolution imagery from Google Earth to remove road segments not constructed by 2000 and 2018. The changes were then digitized. We additionally used a road weighting scheme (i.e., weights for trunk, primary and secondary/tertiary roads were 100, 50, and 25 respectively) to reflect the varying degrees  to which different road classes attract urban growth in the SLEUTH algorithms; minor roads (e.g., secondary/tertiary) have a local effect on urbanization, while major roads (e.g., trunk) allow urbanization to occur further away from the road network. All data processing was conducted using the QGIS tool (available at https://qgis.org/).

#### Calibration and prediction

SLEUTH uses the historical data input to calibrate a set of five coefficients (dispersion, breed, spread, slope resistance, and road gravity) that control the system's behavior. All five coefficients are integers ranging from 0 to 100. The magnitude of the coefficient values determines the extent to which each of four growth rules (spontaneous, new-spreading centers, edge, road-influenced) influence urban growth within the system. Additionally, a set of meta-level rules, named "self-modification" rules, respond to the overall growth rate and change the coefficient values during rapid or slow growth periods^64^. While some efforts (e.g., use of genetic algorithms) have been made to enhance the computational efficiency of SLEUTH model calibration, we employed the standard calibration process known as "brute force calibration". During this procedure, the values of the control coefficients are refined in three sequential phases (coarse, fine, final). Previous studies have used the Lee-Sallee statistic and the Optimal SLEUTH Metric (OSM)^80^ to determine the best-fit coefficients (i.e., those that produce a simulated map most closely resembling the control data) from calibration. In line with current practices, we employed the OSM metric to provide the most robust results. This metric is calculated as the product of seven statistics reported by SLEUTH (compare, population, edges, clusters, slope, X-mean, and Y-mean), each ranging from 0 to 1.

We resampled all the SLEUTH inputs to two spatial resolutions (60 m, 30 m), using the nearest neighbor method. The 60 m-resolution inputs were employed in the coarse calibration while the 30 m-resolution inputs were used in both the fine and the final calibration. During the coarse calibration, the five coefficients were set between 0 and 100, with a step value of 25. Based on these values, the model tried every possible permutation of the five coefficients (a total of 3,125)  in multiple runs. The three sets of best-fit coefficients (i.e., those with the highest OSM) from the coarse calibration were used to initiate the next sequences of permutations (a total of 4500) for the fine calibration, over a narrowed coefficient space and with a smaller step value. Similarly, the three best-fit coefficients from the fine calibration were used to begin the final calibration (2,500 permutations). The combination of best-fit coefficients from the final calibration phase was then used to run SLEUTH over the calibration period (1975–2018) and obtain updated coefficient values corresponding to the final year of model calibration. The updated coefficient values were averaged in a Monte Carlo process and the results were used to initiate urban growth forecasting to 2050. We used 100 Monte Carlo iterations for this process; average coefficients do not vary significantly with more iterations.

The SLEUTH outputs consist of a series of annual probability-of-urbanization maps (30 m resolution), based on 100 Monte Carlo iterations (note that probabilities do not vary significantly with more iterations). As proposed by other studies^19,62,63,70^, we reclassified the probabilities into binary (i.e., urban/non-urban) outcomes using a probability-of-urbanization threshold of 50% to produce the final urbanization maps.

#### Validation

To validate urban growth forecasting of the SLEUTH model, we conducted an accuracy assessment of the predicted map for 2021, using two map comparison metrics: K_simulation_ and its components (K_transition_, K_Transloc_)^81^, and quantity and allocation disagreements ^82^. A detailed description of the map comparison metrics is provided in the Appendix section. The validation was performed using the Map Comparison Kit tool^83^. We considered the observed map of 1975 (the earliest urban map) as the original map to compute K_simulation_ and its components. In addition, we created the observed map of 2021 (against which the predicted map is compared) in the QGIS tool, by appropriately modifying the 2014 urban map (not used in the SLEUTH model) from the GHS-BUILT-R2018A dataset. The label (i.e., “urban/non-urban”) of each pixel in GHS-BUILT-R2018A was verified according to the presence of buildings in current-day high-resolution imagery from Google Earth. Some pixel labels were also edited for consistency with the excluded map.

### Social vulnerability assessment with the SoVI

Social vulnerability and resilience have emerged as core concepts to describe the capacity of social systems to prepare, absorb, adapt and recover from the effects of natural hazards^22,84,85^. The severity of these effects can be disproportionally larger for some population groups (e.g., certain communities within a region). In addition, the underlying socio-economic and demographic characteristics (e.g., gender, age, income, access to education and health services) of a community influence their social vulnerability^86^. However, traditional risk-quantification methods often do not assess people vulnerability or assume a homogeneous vulnerability of the entire population^87^. The inclusion of social vulnerability in natural-hazard risk assessment can be beneficial for policymakers in developing tailored risk reduction strategies, particularly targeting the most vulnerable and marginalized.

Different methods can be employed to quantify social vulnerability to natural hazards. The most frequently used methods are based on composite indicators, such as the Human Development Index^88^, the Prevalent Vulnerability Index^89^, or the Social Vulnerability Index^22^. These indicators are quantitative metrics that enable places to be compared and their corresponding vulnerability trajectories to be tracked. Additionally, these indicators are relatively easy to interpret for non-experts. The aforementioned attributes make composite indicators attractive for policymaking and public risk communication^90^. For instance, the Global Social Vulnerability Map^91^ released by GEM uses a composite index to explain why some countries will experience adverse impacts from earthquakes differently. The social vulnerability index (SoVI) remains the leading conceptual framework to assess social vulnerability^84,92^. This method was formulated to evaluate the social vulnerability of United States’ counties to natural hazards. In its current configuration, the SoVI is calculated based on 29 socio-economic variables that are placed into a principal component analysis to derive a smaller set of statistically optimized components (e.g., wealth, race and social status, age).

Most resilience and social vulnerability frameworks were established in industrialized, high-income nations, which makes their application in other contexts (e.g., low-income countries) challenging or even infeasible. This has led many researchers to modify the standard conceptual frameworks to account for the specific characteristics of their study regions. The main modifications include adding new dimensions of social vulnerability or removing inappropriate ones, adapting the variables selected to represent dimensions, and placing various weights on the different dimensions^84^. In this way, the SoVI methodology has been modified by many scholars and applied in several geographical and social contexts^23,93–99^.

#### Selection of variables and indicators

SoVI uses distinct variables to represent relevant social vulnerability indicators (or dimensions). We employed 11 variables collected from the most recent National Population and Housing Census 2011^28^. Our analysis used Nepal’s former administrative division units (i.e., municipalities and VDCs), given the census date. The selected variables contain demographic and socio-economic attributes of Kathmandu Valley’s population that, according to the literature, influence their social vulnerability to natural hazards. The variables were placed into one of five indicators—Population, Education, Economy, Habitat, and Infrastructure—and we calculated an average vulnerability score per indicator, following the approach proposed by Rodriquez et al.^23^. (Thus, we did not perform a principal component analysis given the narrow set of selected variables).

Table 1 provides the list of variables and indicators used to construct the SoVI for Kathmandu Valley. The Population indicator is composed of three variables. Young children (under 14 years old), older adults (over 60 years old), and individuals with physical or mental disabilities are commonly regarded as the most vulnerable members of the population. In addition, women are seen as more vulnerable than men due to their sector-specific employment, lower salaries and family care responsibilities^22^. The Education indicator is composed of a single variable. Education leads to a better-informed population and a higher level of preparedness for disasters. In contrast, illiterate populations have more difficulties understanding warning information and accessing recovery information^22^. The Economy indicator is composed of three variables. More wealth increases possibilities to absorb and recover from losses, through insurance, social safety nets, and government assistance^22^. As the census does not provide information on household income or consumption, we selected access to mobile/telephone services, mass media communication (TV, radio, internet) and means of transportation (motorcycle, cycle, others) as proxies for wealth. The lack of access to these services/assets indicates lower economic well-being. The Habitat indicator is composed of two variables. Urban areas with high population density can be more challenging to evacuate after a disaster. Also, households with large families often have limited resources and must balance work with family responsibilities^22^. Finally, the Infrastructure indicator is composed of two variables. Access to critical facilities can enhance emergency response after a disaster. In contrast, a lack of access to basic services, such as sanitary facilities and electricity, may compromise health and safety during disaster recovery.

**Table 1:** Indicators and variables of the Social Vulnerability (SoVI) Index.

Indicator		Variable	
No.	Name	No.	Description
1	Population	1	Percentage of people under age 14 and over age 60
		2	Percentage of people with physical or mental disability
		3	Percentage of women
2	Education	4	Percentage of illiterate population aged 5 and older
3	Economy	5	Percentage of households with no mobile phone or telephone service
		6	Percentage of households with no access to at least one means of mass media communication (TV, radio, internet)
		7	Percentage of households with no access to at least one means of transportation (motorcycle, cycle, others)
4	Habitat	8	Population density (hab/km^2^)
		9	Average number of people per household
5	Infrastructure	10	Percentage of households with no toilet facility
		11	Percentage of households with no electricity

Two previous applications of the SoVI in Nepal^97,99^ and one application in Nablus (Palestine)^23^ inspired our selection of variables and indicators. Note that both previous SoVI studies of Nepal differ significantly to our work in terms of scale; we conducted a detailed regional assessment (in Kathmandu Valley), whereas Gautam^99^ and Aksha et al.^97^ examined social vulnerability on a more coarse, national scale. We employed most variables used by Gautam^99^, excluding only two (i.e., percentage of female-headed households with no shared responsibility, population change 2000–2010) that describe similar information to other variables (i.e., percentage of women, population density). We used only a small sub-group of the variables leveraged in the study of Aksha et al.^97^. We excluded some of their variables on house construction materials (e.g., percentage of households without reinforced cement concrete foundation, percentage of population living in houses with low-quality external walls), which are more appropriate for assessing physical vulnerability. We also neglected some social dimensions (e.g., Ethnicity, Migration, Renters), given the more local context of our study, and characterized others (e.g., Education, Economy) with less variables. Due to limited data availability, we did not include three indicators (i.e., Health, Governance and Institutional Capacity, Awareness) proposed by Rodriquez et al.^23^ in our analysis.

#### Step-by-step calculation of the social vulnerability index

The SoVI is a relative measure of the social vulnerability of one spatial unit compared to others. The SoVI of each municipality/village was calculated according to the following four steps.

*Step 1*: Each variable (${\mathrm{V}}_{\mathrm{m}}$) value was converted to a normalized version (${\mathrm{NV}}_{\mathrm{m}}$) expressed in a standard scale, where 0 and 1 indicate the least and the most vulnerable values, respectively. The normalization procedure consisted of comparing each variable to the corresponding minimum (${\mathrm{min }}_{\mathrm{m}}$) and maximum values (${\mathrm{max }}_{\mathrm{m}}$) of the total study area, as follows:

1 $${\mathrm{NV}}_{\mathrm{m}}=\frac{{\mathrm{V}}_{\mathrm{m}}-{\mathrm{min }}_{\mathrm{m}} }{{\mathrm{max }}_{\mathrm{m}}-{\mathrm{min }}_{\mathrm{m}}}$$

*Step 2*: The score per indicator (${\mathrm{I}}_{\mathrm{n}}$) was computed as the arithmetic mean of the corresponding ${\mathrm{NV}}_{\mathrm{m}}$ values.

*Step 3*: The overall SoVI was calculated by summing each ${\mathrm{I}}_{\mathrm{n}}$, i.e.:

2 $$\mathrm{SoVI}={\mathrm{I}}_{1}+{\mathrm{I}}_{2}+{\mathrm{I}}_{3}+{\mathrm{I}}_{4}+{\mathrm{I}}_{5}$$

This implies that an equal weighting for all indicators was assumed, in line with typical applications of the SoVI^90^. In addition, we performed a sensitivity analysis that showed no significant variations in the results (reported in Table 4) for alternative weighting schemes.

*Step 4*: The SoVI scores were classified into five categories (very low, low, moderate, high, and very high) using a quantile classification (i.e., each category contains an equal number of values).

## Results and discussion

### Urban growth calculations

#### Changes in built-up areas over time

**Figure 4:**
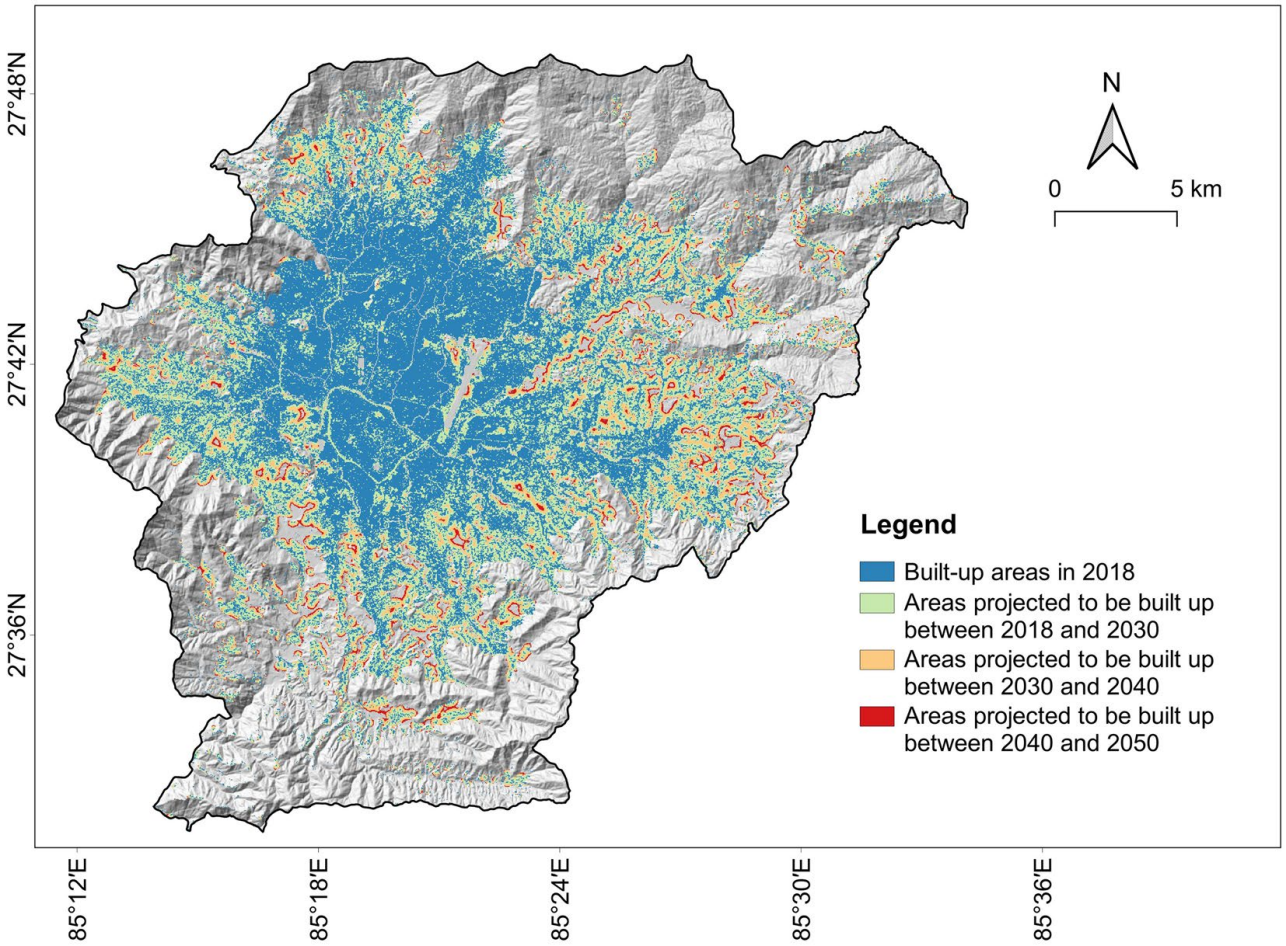
Existing built-up areas in Kathmandu Valley (as of 2018) and projected built-up areas by 2050.

Table 2 summarizes changes in Kathmandu Valley's built-up areas from the past (1975) to the present (2018) and future (classified in three epochs up to 2050). It can be observed that the built-up areas in Kathmandu Valley have dramatically increased from 41 km^2^ to 177 km^2^ between 1975 and 2018. This rapid urban growth is in good agreement with land-use changes estimated by the local authorities^100^, which indicates that the historical urban maps are reasonably accurate. Moreover, built-up areas are predicted to reach 352 km^2^ by 2050, almost doubling their current size and covering about 50% of Kathmandu Valley's land area. Additionally, the pace of urbanization observed from 2000 to 2018 (annual growth rate of 6.9%) will remain intense until 2030 (annual growth rate of 4.5%). After 2030, urban expansion will gradually slow down, reflecting the typical S-curve growth of urbanization. The spatial distribution of existing built-up areas until 2018 and predicted built-up areas by 2050 are shown in Fig. 4.

**Table 2:** Changes in Kathmandu Valley’s built-up areas over time.

Name	1975	1990	2000	2018	2030	2040	2050
Built-up area (km^2^)	41	46	53	177	301	342	352
Urban land percentage (%)	5.7	6.4	7.4	24.6	41.7	47.4	48.8
Annual growth rate (%)		0.8	1.4	6.9	4.5	1.3	0.3

$$\mathrm{Annual \,\,growth \,\,rate }={\left(\frac{{\mathrm{A}}_{\mathrm{j}}}{{\mathrm{A}}_{\mathrm{i}}}\right)}^{1/\Delta \mathrm{t}}-1$$.

$${\mathrm{A}}_{\mathrm{i}}=\mathrm{built \,\,up\,\, areas \,\,at \,\,the \,\,starting \,\,year}$$, $${\mathrm{A}}_{\mathrm{j}}=\mathrm{built \,\,up \,\,areas \,\,at \,\,the \,\,end \,\,year}$$, $$\Delta \mathrm{t}=\mathrm{number \,\,of\,\, years}$$^19^.

#### Calibration and validation of SLEUTH

The set of best-fit coefficients obtained during the calibration stage (at the start year of model calibration) was Diffusion = 22, Breed = 90, Spread = 27, Slope = 68, Road-gravity = 15. The goodness-of-fit statistics for this combination were compare = 0.88, population = 0.95, edges = 0.97, clusters = 0.99, slope = 0.99, X-mean = 0.93, and Y-mean = 0.83. The product of the previous seven metrics results in an OSM of 0.62, which indicates a good performance of SLEUTH in capturing the urbanization trends in the study area. This value is higher than that reported in other applications of SLEUTH for urban areas in the United States (0.25)^61^, Italy (0.40)^65^, China (0.48)^63^, and Iran (0.49)^66^. The high value of Breed shows that the emergence of new-spreading centers has been the major  source of urban growth in Kathmandu Valley over the past decades. The lower values of Diffusion, Spread and Road-gravity show less participation of spontaneous growth, edge growth and road-influenced growth in the dynamics of urban expansion. Moreover, the high value of Slope reflects that topography has played a key role in controlling urbanization across the valley.

**Figure 5:**
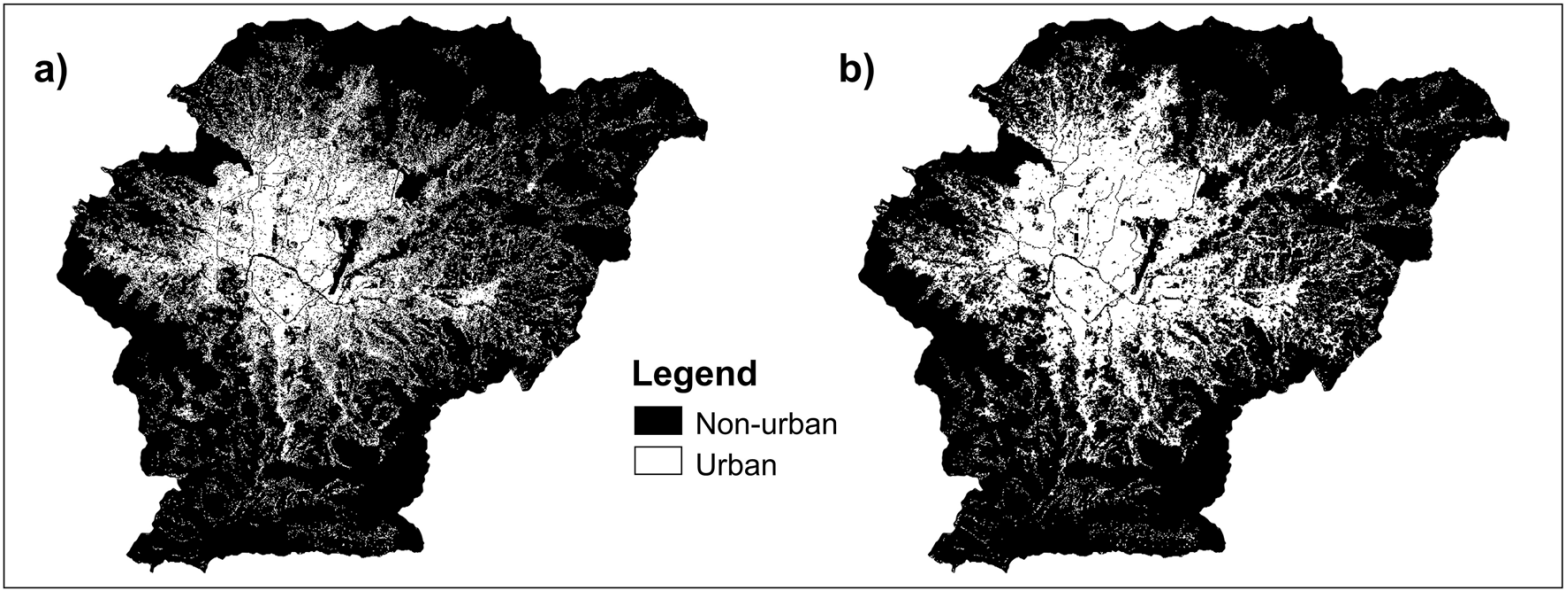
(**a**) Observed; and (**b**) Predicted urbanization maps of Kathmandu Valley for 2021.

The observed and predicted maps of 2021 used for the SLEUTH validation exercise are shown in Fig. 5. The K_simulation_ was calculated as 0.62, which offers an acceptable level of total accuracy. To provide some context, a previous study that assessed the performance of four LULC models (including SLEUTH) in simulating future urban growth in Charlotte, North Carolina^101^ reported K_simulation_ values below 0.51, while an application of SLEUTH for Gorizia, Italy^67^ produced K_simulation_ values ranging between 0.71 and 0.91. We obtained a K_Transition_ value of 0.83 and a K_TransLoc_ value of 0.74 in this study, which indicate that the amount of urban area was slightly better predicted than its location across the landscape. This conclusion is also supported by the values of quantity disagreement and allocation disagreement, which were 5.1% and 7.3% respectively. Overall, the predicted map was found to overestimate the amount of urban area by 22%. This overestimation, however, does not depend solely on the performance of SLEUTH but is highly influenced by the seed map used to initiate the prediction. The 2018 seed urban map accounts for 177 km^2^ of urban area, which is larger than the 165 km^2^ depicted in the 2021 observed map. This means that at least 7% of the 22% overestimation can be attributed to inaccuracies with the input data. Although the best available data on Kathmandu Valley's urban cover was used, these issues underline the importance of selecting accurate data inputs when simulating urban growth. Furthermore, the reasonably high value of OSM suggests that the model can simulate future urban growth with some confidence.

### Social vulnerability calculations

**Figure 6:**
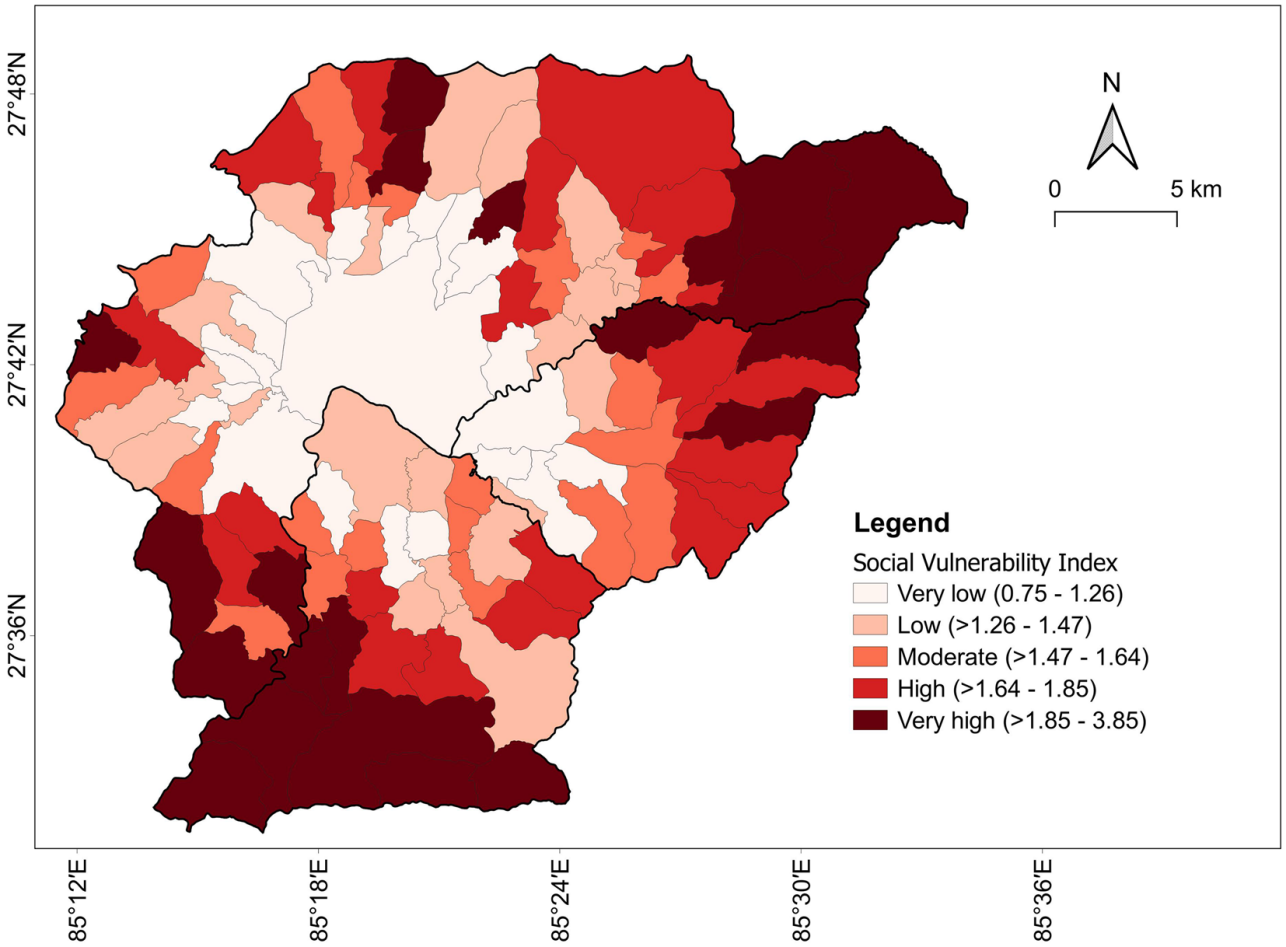
SoVI map.

**Figure 7:**
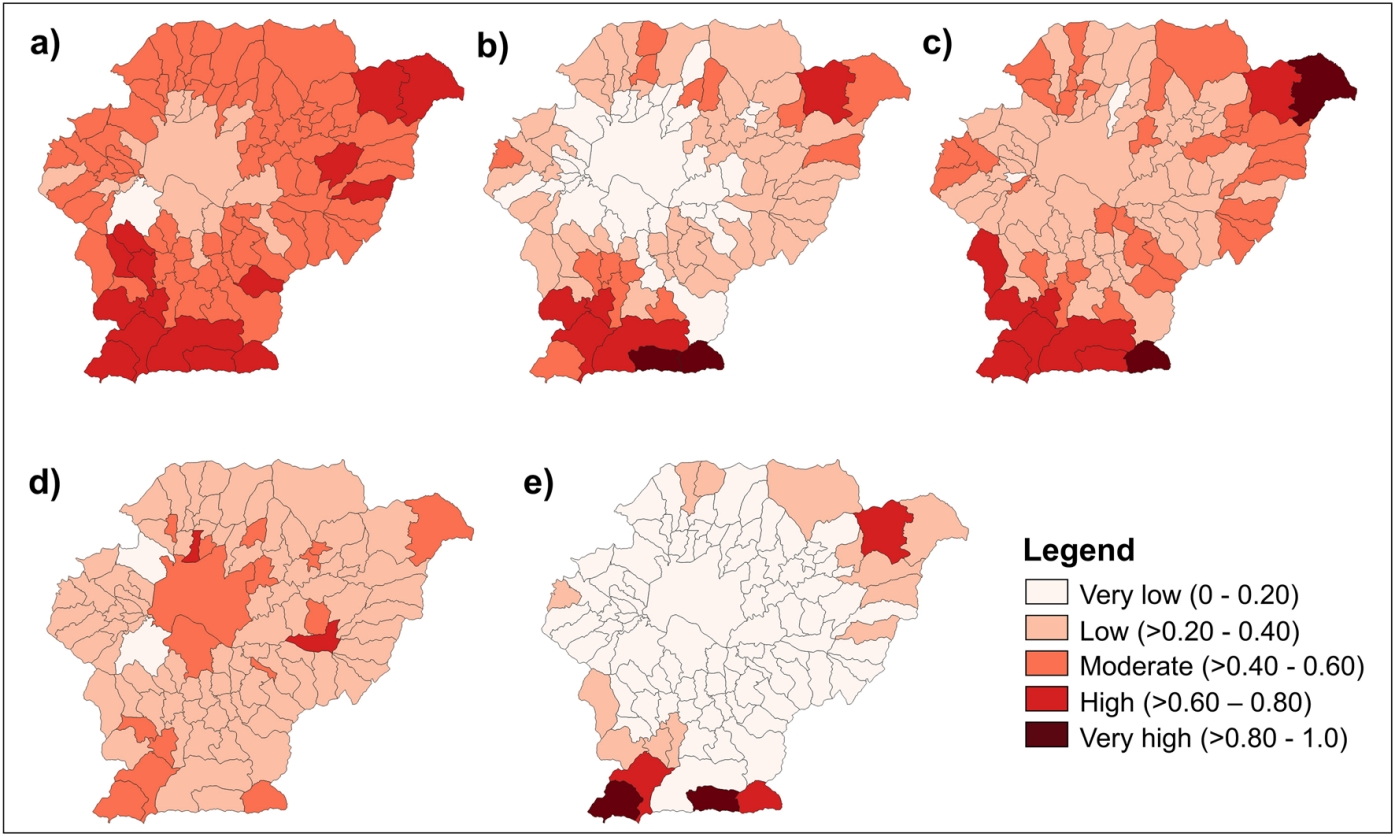
Social vulnerability score per indicator (${\mathrm{I}}_{\mathrm{n}})$: (**a**) Population; (**b**) Education; (**c**) Economy; (**d**) Habitat; and (**e**) Infrastructure.

We used socio-demographic data to calculate the SoVI in 104 municipalities/villages of Kathmandu Valley. Table 3 presents an overview of the values obtained for the 11 variables used in the analysis. We note that dispersion is considerable in some variables (e.g., 8: population density, 10: percentage of households with no toilet facility, 11: percentage of households with no electricity). These variations suggest that even though Kathmandu Valley is perceived as the most developed region in Nepal, inequalities persist at local levels. Figure 6 displays the SoVI scores for each municipality/village based on percentile ranks. As shown in the map, the level of social vulnerability is not uniform. Most locations in the central, less elevated part of the valley have very low or low vulnerability. In contrast, many areas on the borders (especially the southern and north-eastern parts) have very high or high vulnerability. Figure 7 illustrates the social vulnerability per indicator (i.e., the ${\mathrm{I}}_{\mathrm{n}}$ value computed in Step 2 from SoVI calculation). Population, Economy and Habitat are the vulnerability indicators that exhibit the lowest variability, with coefficients of variation of 20%, 36% and 24%, respectively. Education and Infrastructure are the most variable vulnerability indicators, with coefficients of variation of 62% and 132%. These ${\mathrm{I}}_{\mathrm{n}}$ values allow us to identify the main drivers of vulnerability for each municipality/village in the region.

**Table 3:** Descriptive statistics of variables used in the social vulnerability analysis.

Indicator	Variable no.	Mean	St. dev	MIN	MAX	Average ${\mathrm{I}}_{\mathrm{n}}$
Population	1	33.0	3.5	14.2	43.9	0.48
	2	1.3	0.8	0.5	6.3	
	3	50.4	1.3	44.4	53.2	
Education	4	21.9	6.9	10.8	48.9	0.29
Economy	5	17.0	8.4	6.6	47.7	0.40
	6	23.3	9.6	6.6	53.3	
	7	73.7	8.8	41.6	97.4	
Habitat	8	3055.4	4192.5	72.3	23,050.9	0.34
	9	4.4	0.3	3.4	5.2	
Infrastructure	10	8.9	14.5	0.0	73.0	0.13
	11	3.4	2.9	0.9	19.1

### Interactions between urban growth, hazard, and social vulnerability

The maps of hazards, urban growth, and social vulnerability were overlaid to investigate their spatial relationships. The analyses focused on projected risk-related trends between the present (2018) and the future (2050). We assumed that current levels of social vulnerability in villages and municipalities will remain unchanged in the future, given the lack of available data to make confident projections. At the same time, past related research suggests that social vulnerability is not expected to vary significantly over time. For instance, Cutter and Finch^102^ reported that 85% of United States' counties showed no statistically significant change in social vulnerability between 1960 and 2000, and only 3% experienced a statistically significant and clear (strong) increase/decrease in vulnerability. Zhou et al.^103^ indicated that only 18% of China’s counties exhibited a significant increase/decrease in social vulnerability between 1980 and 2010. Frigerio et al.^104^ found that the percentage of Italian municipalities per social vulnerability class (very low, low, medium, high, very high) showed only small variations (from 2.9% to 8.8%) between 1991 and 2011.

**Figure 8:**
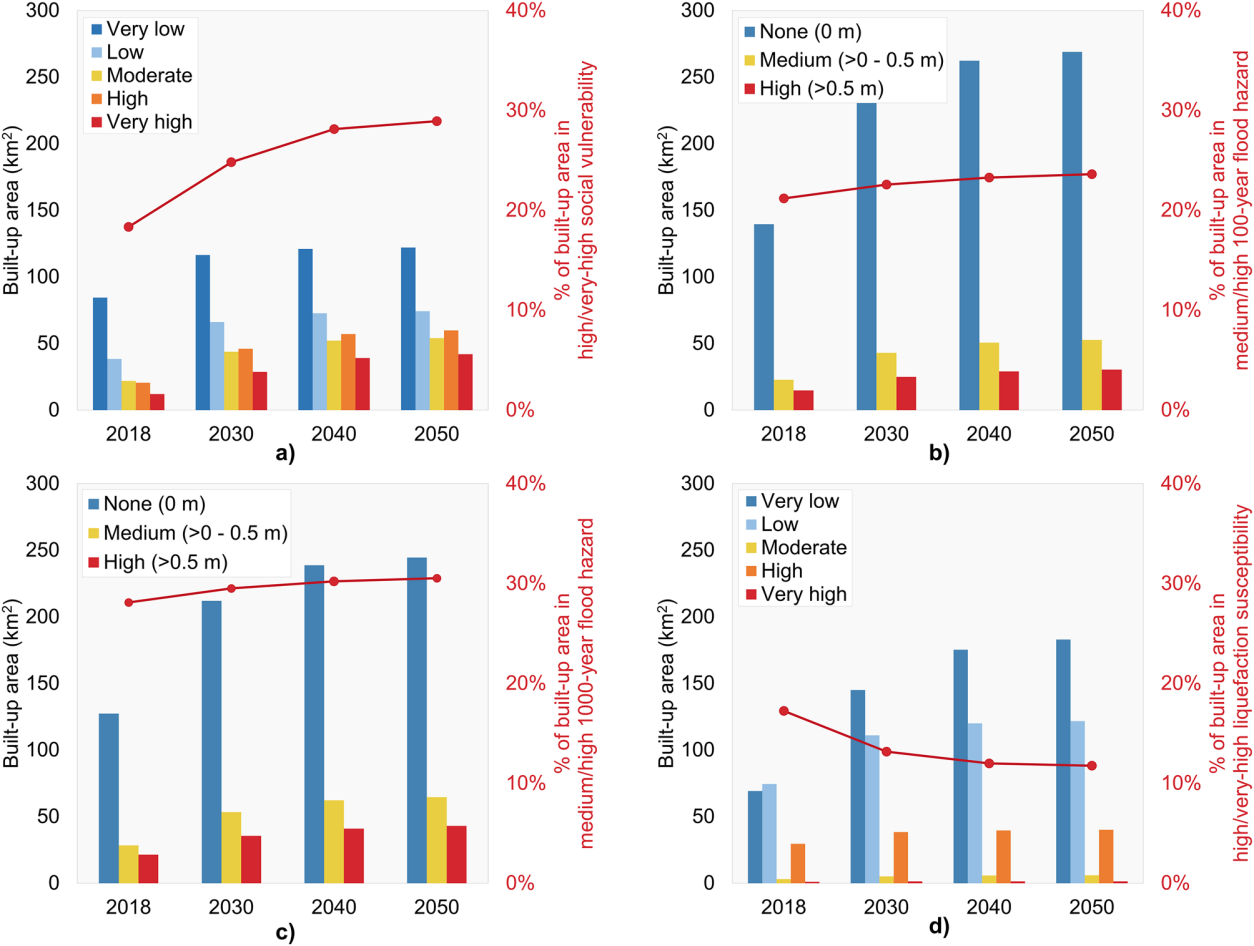
Evolution in built-up areas disaggregated by (**a**) SoVI category; (**b**) 100-year flood hazard depth range; (**c**) 1000-year flood hazard depth range; and (**d**) Liquefaction susceptibility level.

Figure 8a and Table 4 (first row) summarize changes in built-up areas, disaggregated on the basis of SoVI category. Over the 2018–2050 period, built-up areas in villages with very low, low, and moderate vulnerability will increase from 145 km^2^ (82% of the total built-up area) to 250 km^2^ (71% of the total built-up area). Over the same period, built-up areas in villages with high and very high vulnerability will increase from 32 km^2^ (18% of the total built-up area) to 102 km^2^ (29% of the total built-up area). While the largest absolute increase in built-up areas (i.e., 105 km^2^) is observed in the less vulnerable (i.e., very low, low, moderate) villages, the contribution of the most vulnerable (i.e., high, very high) villages to the urban structure of Kathmandu Valley will increase by 11%. In addition, urbanization will occur more rapidly in places with higher vulnerability. For instance, from 2018 to 2030, villages with very high vulnerability will experience an annual growth rate of 7.6%, almost three times more than the annual growth rate of 2.7% for villages with very low vulnerability. The relative difference in growth rate is projected to be even more significant over the subsequent 2030–2040 and 2040–2050 periods (see Table 4).

**Table 4:** Changes in built-up areas disaggregated by SoVI category, flood hazard depth range, and liquefaction susceptibility level.

Vulnerability/hazard level	Built-up area (km^2^)				Share of built-up area (%)				Annual growth rate (%)		
	2018	2030	2040	2050	2018	2030	2040	2050	2018–2030	2030–2040	2040–2050
**SoVI**											
Very low	85	116	121	122	47.7	38.7	35.4	34.6	2.7	0.4	0.1
Low	38	66	73	74	21.6	21.9	21.2	21.1	4.6	1.0	0.2
Moderate	22	44	52	54	12.3	14.6	15.3	15.3	6.0	1.7	0.3
High	21	46	57	60	11.6	15.3	16.7	17.0	7.0	2.2	0.5
Very high	12	29	39	42	6.8	9.5	11.4	11.9	7.6	3.1	0.7
**100-year flood hazard**											
None (0 m)	140	233	262	269	78.8	77.4	76.7	76.4	4.4	1.2	0.2
Medium (> 0–0.5 m)	23	43	51	53	12.9	14.3	14.8	15.0	5.4	1.6	0.4
High (> 0.5 m)	15	25	29	30	8.3	8.3	8.5	8.6	4.5	1.5	0.5
**1000-year flood hazard**											
None (0 m)	127	212	239	245	71.9	70.5	69.8	69.5	4.3	1.2	0.2
Medium (> 0–0.5 m)	28	53	62	65	16.0	17.8	18.2	18.3	5.4	1.5	0.4
High (> 0.5 m)	21	35	41	43	12.1	11.8	12.0	12.2	4.3	1.5	0.5
**Liquefaction susceptibility**											
Very low	69	145	175	183	39.0	48.2	51.3	52.0	6.4	1.9	0.4
Low	74	111	120	122	42.0	36.9	35.1	34.6	3.4	0.8	0.2
Moderate	3	5	6	6	1.7	1.7	1.7	1.7	4.3	1.2	0.2
High	30	38	40	40	16.7	12.7	11.6	11.4	2.2	0.3	0.1
Very High	0.9	1	1	1	0.5	0.4	0.4	0.4	2.7	0.1	0.0

$$\mathrm{Annual \,\,growth \,\,rate }={\left(\frac{{\mathrm{A}}_{\mathrm{j}}}{{\mathrm{A}}_{\mathrm{i}}}\right)}^{1/\Delta \mathrm{t}}-1.$$

$${\mathrm{A}}_{\mathrm{i}}=\mathrm{built \, up \, areas \, at \, the \, starting \, year}$$, $${\mathrm{A}}_{\mathrm{j}}=\mathrm{built \, up \, areas \, at \, the \, end \, year}$$, $$\Delta \mathrm{t}=\mathrm{number \, of \, years}$$^19^.

Figure 8b and Table 4 (second row) provide the changes in built-up areas disaggregated by 100-year flood hazard depth range. By 2050, built-up areas exposed to each 100-year flood-depth range will nearly double their current size. Over the 2018–2050 period, built-up areas exposed to the lowest flood depth (i.e., non-inundated areas) will expand from 140 km^2^ (79% of total built-up area) to 269 km^2^ (77% of total built-up area). Also, built-up areas exposed to moderate and high 100-year flood-depth ranges (i.e., inundated areas) will increase from 38 km^2^ (21% of total built-up area) to 83 km^2^ (23% of total built-up area). Figure 8 (panel c) and Table 4 (third row) present the changes in built-up areas disaggregated by 1000-year flood hazard depth range. Over the 2018–2050 period, built-up areas in non-inundated regions will increase from 127 km^2^ (72% of total built-up area) to 245 km^2^ (70% of total built-up area). This means that built-up areas in inundated regions will increase from 49 km^2^ (28% of total built-up area) to 108 km^2^ (30% of total built-up area). Therefore by 2050, there is projected to be 25 km^2^ more built-up area in the inundated region of the 1000-year flood hazard map than in that of the 100-year hazard map. The projected annual urban growth rates for both flood hazard levels do not show significant differences.

Figure 8d and Table 4 (fourth row) provide changes in built-up areas per liquefaction susceptibility level. It is noticeable that urban growth will occur almost exclusively in areas with low levels of liquefaction susceptibility. Over the 2018–2050 period, built-up areas in locations with very low and low susceptibility will increase from 143 km^2^ (81% of total built-up area) to 295 km^2^ (87% of total built-up area). Also, built-up areas in locations with moderate, high, and very high susceptibility will only increase from 34 km^2^ (19% of total built-up area) to 47 km^2^ (13% of total built-up area). These trends can be explained by the fact that the highest liquefaction susceptibility is predominantly associated with the central part of the valley, where there is minimum land available for urbanization.

## Conclusions

This paper has examined spatial relationships between natural hazards, urban growth, and social vulnerability in Kathmandu Valley, Nepal. Two widely used methods, the cellular-automata SLEUTH model and the composite Social Vulnerability Index (SoVI), have been implemented to simulate future urban expansion and quantify social vulnerability, respectively. In addition, two pre-existing datasets have been employed to characterize the extent and severity of flooding and liquefaction in the region. The combination of urban growth estimates with hazard values and social vulnerability indicators provide evidence to support policymaking in disaster risk management.

Results show that Kathmandu Valley will continue expanding fast and intensively until 2030 and at a decreased pace until 2050. By the mid-century, the total extent of built-up areas will reach 352 km^2^, nearly doubling their current size and covering half the entire valley. In addition, 29% of the total built-up area in 2050 will be located in the most vulnerable villages, which is 11% more than the present proportion of urbanization associated with these areas. Moreover, for a 100-year flood hazard, 83 km^2^ of the total urban area in 2050 will be distributed in potentially inundated zones, which is twice the current amount. For a more severe 1000-year flood hazard, the total built-up area flooded regions will reach 108 km^2^ by 2050. Furthermore, built-up areas susceptible to liquefaction will total 47 km^2^ by 2050, 13 km^2^ more than their current size.

The  notable increase in built-up areas projected to occur in the most vulnerable and hazardous regions of Kathmandu Valley emphasizes the critical importance of policymaking for shaping a sustainable future. The enormous land available for urbanization suggests that land-use regulations can effectively control future exposure to natural hazards and limit potential disaster losses. For instance, urban development should be prevented or tightly constrained in high-hazard areas, while new urbanization and relocation should be promoted in low-hazard zones. At the same time, it is interesting to note that  significant future expansion is predicted to take place away from the most hazardous locations of Kathmandu Valley: 129 km^2^ and 162 km^2^ of new urban area in 2050 will be respectively distributed in potentially non-inundated (for a 100-year flood hazard) and non-liquefiable areas. This spatial trend between natural hazards and urban expansion is not due to an implicit feature of the urban growth model used in this study. (One could even expect the opposite trend, since SLEUTH’s transition rules consider higher likelihood of urbanization for low-slope land that is indirectly related with high liquefaction susceptibility and high flood hazard.) Rather, the prediction of large future expansion away from hazardous areas is driven by the constraints of past planning decisions, which have already urbanized most land in the central (and most hazardous) part of the valley. In addition to encouraging risk-sensitive land-use planning, recognizing vulnerable groups and identifying the main drivers of social vulnerability can assist decision-makers in designing individual soft (e.g., insurance) and hard policies (e.g., retrofitting schemes, building-code enhancement) for disaster risk reduction.

Finally, while this paper focuses on the interactions of urban growth with flooding and liquefaction in Kathmandu Valley, similar analyses can be conducted in other geographical regions to assess the impact of different natural hazards (e.g., landslides, wildfire) on future exposure and its spatial relationship with vulnerability. Moreover, updated census information and data improvements can be employed to adjust the estimations of social vulnerability used in this study and even to produce future projections of social vulnerability. While previous research suggests that social vulnerability is not expected to vary significantly over a relatively short timeframe (such as the one considered in this study), forecasting social vulnerability for disaster risk assessments remains a major challenge. Potential changes in social vulnerability could occur gradually (e.g., influenced by population trends, such as demographic skewing toward the elderly or very young) or almost instantaneously (e.g., in response to natural-hazard events), and can affect the number of casualties, the loss or disruption sustained, and a community’s subsequent recovery time^14^. In addition to the valuable insights provided by social vulnerability analysis, the inclusion of physical vulnerability in the calculations is required to quantify the extent of losses sustained by built structures and more holistically measure the effectiveness of risk-reduction strategies. Further improvements can be made to consider the effects of urbanization on flood hazard (i.e., increased runoff during rainfall events), which were not accounted for in the hazard maps used in this study. Lastly, note that the results presented here are estimated assuming a business-as-usual scenario, which considers future urban expansion to be an extension of historical urban growth. The inclusion of environmental considerations (e.g., preventing forest area from future urbanization), plans for infrastructure development (e.g., new roads), or strategies for land-use management (e.g., zoning plans) in the analysis can result in alternative future urban growth pathways. Future research should focus on exploring these alternative scenarios and possibly examine the feasibility of incorporating a two-way feedback loop approach that would allow the risk associated with one urban scenario to be used to constrain more risk-informed urban growth pathways. Some attempts towards this aim have already been made in the literature^105^, but for more simplified urban growth scenarios. Moreover, despite the numerous advantages of cellular-automata models for urban growth modelling, there are also some drawbacks. The main limitations are the lack of a clear theoretical link between the transition rules and the actual agents of decision-making, and the models’ scale dependency (i.e., results can be sensitive to cell size and neighborhood configuration)^59^. Some current challenges for urban cellular-automata models include accounting for the multi-dimensional processes of urban change (e.g., urban regeneration, densification and gentrification, vertical urban growth), and benefitting from emergent sources of big data to calibrate/validate models and capture the role of individual human decision behaviour^106^. Integrating cellular-automata models with other techniques (e.g., multi-agent systems, Markov-Chain algorithms, regression models) can help to provide more robust information for informed urban planning.

## Supplementary Information

Supplementary Information.

## Data Availability

The datasets used and/or analyzed during the current study are available from the corresponding author on reasonable request.
