# Supplementary material for: Urban growth modelling and social vulnerability assessment for a hazardous Kathmandu Valley
Source: Sci Rep. 2022 Apr 12;12:6152. doi: 10.1038/s41598-022-09347-x (PMC9005627; doi:10.1038/s41598-022-09347-x)
Supplement: Supplementary file 1 — Supplementary Information. [file 41598_2022_9347_MOESM1_ESM.docx]

Supplementary Information

**Urban growth modelling and social vulnerability assessment for a hazardous Kathmandu Valley**

Carlos Mesta^1,*^, Gemma Cremen^2^, Carmine Galasso^1,2^

^1^ UME Graduate School, Scuola Universitaria Superiore IUSS Pavia, Pavia, Italy

^2^ Dept. of Civil, Environmental and Geomatic Engineering, University College London, London, United Kingdom

* Corresponding author, email: carlos.mesta@iusspavia.it

1. **Appendix**

**Metrics used in the validation of urban growth forecasting**

Traditional metrics, such as the Kappa statistic, express the agreement between two categorical maps corrected for the expected agreement that is based on a stochastic model of random allocation given the distribution of class (i.e., urban/non-urban) sizes (i.e., the fractions of cells in each class). However, the distribution of class sizes is not a meaningful reference level for models that start from an original land-use map. To overcome these issues, we used a modified version of the Kappa statistic K_simulation_ that adjusts the expected agreement by the information in the original land-use map ^1^. Thus, the K_simulation_ statistic and its components (K_transition_, K_Transloc_) report the agreement between the observed and predicted land-use maps while accounting for land-use persistence (i.e., the fact that most locations do not change their original land-use).

K_simulation_ and its components can be calculated as follows:

$K_{\mathrm{Simulation}}=\frac{p_{o}-p_{e_{\left( \mathrm{transition} \right)}}}{1-p_{e(transition)}}$ (Equation 1)

$K_{\mathrm{Transition}}=\frac{p_{max(transition)}-p_{e(transition)}}{1-p_{e(transition)}}$ (Equation 2)

$K_{\mathrm{Transloc}}=\frac{p_{o}-p_{e(transition)}}{p_{max(transition)}-p_{e(transition)}}$ (Equation 3)

where K_transition_ reports the agreement in the quantity of land-use changes, K_Transloc_ reports the degree to which the changes agree in their allocations, p_o_ is the observed proportion of agreement. p_e(transition)_ is the expected fraction of agreement given the size of class transitions, i.e.:

$p_{e_{\left( \mathrm{transition} \right)}}=\sum_{c=1}^{j} p\left( o=j \right)\cdot\sum_{c=1}^{i} p\left( a=i | o=j \right)\cdot p\left( s=i | o=j \right)$ (Equation 4)

where $p\left( a=i | o=j \right)$ is the fraction of pixels that changed from class “j” in the original map (O) to class “i” in the predicted map A, $p\left( s=i | o=j \right)$ is the fraction of pixels that changed from class “j” in the original map (O) to class “i” in the observed map (S).

p_max(transition)_ is the maximum accuracy that can be achieved given the size of the class transitions:

$p_{max(transition)}=\sum_{c=1}^{j} p\left( o=j \right)\cdot\sum_{c=1}^{i} \min\left( p\left( a=i | o=j \right), p\left( s=i | o=j \right) \right)$ (Equation 5)

where all variables are as previously defined.

The values of K_simulation_ and K_Transloc_ ranges from -1 to 1, where 1 indicates perfect agreement, 0 indicates chance agreement due to random distribution of the given class transitions, and below 0 indicate less accuracy than chance agreement due to random distribution. The values of K_transition_ range from 0 to 1, where 0 indicates that there are no class transitions that appear in both maps, and 1 indicates perfect agreement in the sizes of class transitions.

Quantity and allocation disagreements are other helpful measures for evaluating discrepancies between the observed and predicted land-use maps. Quantity disagreement expresses the amount of difference between the two maps due to the relative match in the proportions of the classes. Allocation agreement describes the amount of difference between the two maps due to the relative match in the spatial allocation of the classes. The total disagreement is the sum of the quantity disagreement and the allocation disagreement ^2^. The quantity disagreement q_g_ and allocation disagreement a_g_ for an arbitrary class can be calculated as follows:

$q_{g}=\left| \left( \sum_{i=1}^{J} p_{\mathrm{ig}} \right)-\left( \sum_{j=1}^{J} p_{\mathrm{gj}} \right) \right|$ (Equation 6)

$a_{g}=2\min\left| \left( \sum_{i=1}^{J} p_{\mathrm{ig}} \right)-p_{\mathrm{gg}}, \left( \sum_{j=1}^{J} p_{\mathrm{gj}} \right)-p_{\mathrm{gg}} \right|$ (Equation 7)

where p_ig_ is the proportion of the study area that is class "i" in the predicted map and class "g" in the observed map, p_gj_ is the proportion of the study area that is class "g" in the predicted map and class "j" in the observed map, and p_gg_ is the proportion of the study area that is class "g" in both predicted map and observed map. By incorporating all "J" classes, the overall quantity disagreement Q and allocation disagreement A can be calculated as follows:

$Q=\frac{\sum_{g=1}^{J} q_{g}}{2}$ (Equation 8)

$A=\frac{\sum_{g=1}^{J} a_{g}}{2}$ (Equation 9)

**References**

1. van Vliet, J., Bregt, A. K. & Hagen-Zanker, A. Revisiting Kappa to account for change in the accuracy assessment of land-use change models. Ecol. Model. **222**, 1367–1375 (2011).

2. Pontius, R. G. & Millones, M. Death to Kappa: birth of quantity disagreement and allocation disagreement for accuracy assessment. Int. J. Remote Sens. **32**, 4407–4429 (2011).
